# Mortality risk during the COVID-19 pandemic is shaped by human development

**DOI:** 10.1186/s44263-026-00255-0

**Published:** 2026-03-02

**Authors:** Kolja Nenoff, Sarah Habershon, Miguel D. Mahecha, Sabine Attinger, Khalil Teber, Guido Kraemer

**Affiliations:** 1 Institute for Earth System Science and Remote Sensing, University Leipzig, Talstr. 35, 04103 Leipzig, Sachsen Germany; 2 Helmholtz Centre for Environmental Research, UFZ, Permoserstr. 15, 04318 Leipzig, Sachsen Germany

**Keywords:** COVID-19, Socioeconomics, Excess mortality, Pandemic preparedness, Explainable AI, Inequality

## Abstract

**Background:**

During the global COVID-19 pandemic (2020–2021), excess mortality varied substantially across countries. Notably, upper-middle-income countries experienced greater variability in excess mortality than both low- and high-income countries, despite reporting fewer COVID-19 cases than high-income countries but more than low-income countries. This disconnect between case numbers and mortality suggests more complex structural vulnerabilities. Socioeconomic conditions and healthcare system performance, collectively referred to as National Framework Conditions (*NFCs*), are likely key determinants of pandemic outcomes. However, the specific relationship between these factors and excess mortality remains poorly understood.

**Methods:**

We constructed a predictive model of excess mortality using reported COVID-19 case counts and a wide array of *NFCs* derived from the World Development Indicators (WDI), employing a tree-based machine learning method (XGBoost). To reduce dimensionality, we applied a non-linear method (e-Isomap), extracting latent components called compressed National Framework Conditions (*cNFCs*). We applied SHapley Additive exPlanations (SHAP) values to estimate the feature importance and quantify the contribution of each *cNFC*.

**Results:**

Our machine learning model explained nearly half of the global variance in excess mortality ($R^2$: median 49.7; interquartile range (IQR): 10.9). SHAP analysis revealed that *cNFCs* contributed most strongly to model predictions of excess mortality (SHAP: median 8.1; IQR 1.2), followed by *pandemic indicators*, such as reported COVID-19 cases (SHAP: median 6.4; IQR 0.7). Using explainable artificial intelligence (XAI), we further identified how interconnected socioeconomic conditions, including labor force participation age and health spending, shaped mortality outcomes.

**Conclusions:**

Our findings demonstrate that *cNFCs* outperform conventional epidemiological or preparedness metrics, in explaining cross-country differences in COVID-19 excess mortality during 2020–2021. By capturing latent socioeconomic structures, the *cNFC* framework reveals systemic vulnerabilities that reported COVID-19 cases and other indicators fail to detect. This approach offers a new perspective on structural resilience and pandemic preparedness.

**Supplementary Information:**

The online version contains supplementary material available at 10.1186/s44263-026-00255-0.

## Background

**Fig. 1:**
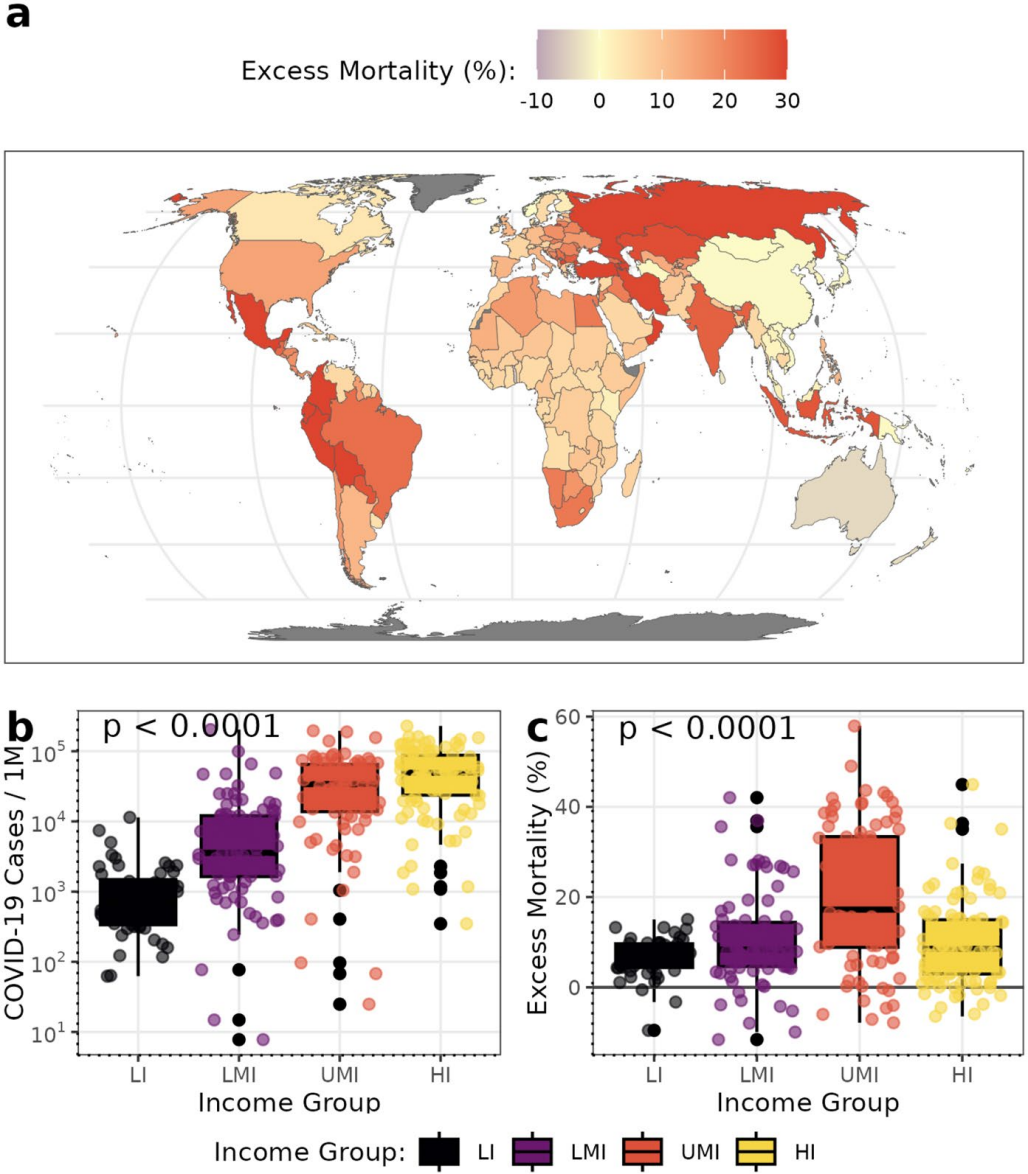
Global Patterns of COVID-19 Cases and Excess Mortality. **a** Illustrates the geographical distribution of mean annual excess mortality for 2020–2021 (capped at 95^th^ percentile for visualization). **b** and **c** show the distribution of COVID-19 cases and excess mortality, respectively, stratified by World Bank income classifications. Statistical differences between income groups were assessed using Kruskal-Wallis tests. Data points in panels **b** and **c** represent country-year observations for 173 countries (*n* = 346 for 2020–2021). Colors correspond to the World Bank country income classifications: low income (LI), lower-middle income (LMI), upper-middle income (UMI), and high income (HI). Data sources: WHO [1] for excess mortality, Our World in Data [6] for COVID-19 cases, and World Bank [28] for income classifications

The COVID-19 pandemic’s impact on excess mortality varied widely across countries and regions, often showing patterns that did not align with reported case numbers. By the end of 2021, the number of deaths directly attributed to COVID-19 reached approximately 5.4 million globally, while estimates of excess mortality ranged from 12 to 22 million [1–5]. These excess deaths were unevenly distributed, with particularly high rates observed in Latin America and countries of the former Soviet bloc, and comparatively lower rates in Oceania and parts of Africa (Fig. 1a).

Reported COVID-19 cases in 2020–2021 followed a gradient corresponding to countries’ income group (Fig. 1b). However, excess mortality in upper-middle-income countries was higher than in low- or high-income countries (Fig. 1c) [6, 7]. The reasons behind cross-country differences in COVID-19 health outcomes remain the subject of ongoing debate [8, 9]. Neither viral exposure measured through reported COVID-19 cases nor containment policies, such as non-pharmaceutical interventions, adequately account for the observed variation in excess mortality across countries [10, 11]. Excess mortality refers to the difference between the total number of observed deaths and the number expected under normal conditions. The World Health Organization (WHO) provides globally harmonized estimates of excess mortality based on this definition, expressed as the percentage difference between observed and expected all-cause deaths (*P*-score excess mortality) [1]. It captures both direct deaths from COVID-19 and indirect deaths arising from the broader effects of the pandemic, such as disruptions to healthcare and social systems [12]. As such, excess mortality provides a more robust and comprehensive measure of the pandemic’s true health outcome [13].

In addition to measuring outcomes, we must account for differences in exposure to the virus, which may confound the relationship between socioeconomic conditions and excess mortality. To represent this exposure, a consistent and globally available covariate is required. Readily accessible indicators include reported COVID-19 cases and deaths, both of which are affected by reporting biases arising from different processes. COVID-19 case data are generally compiled using simpler and more standardized criteria, primarily reflecting testing and surveillance capacity, whereas COVID-19 death data depend on cause-of-death certification, medical review, and registration systems that vary widely across countries. The question of how to measure population exposure remains a topic of ongoing debate, with different approaches involving trade-offs between data availability and comparability across countries [14–18]. We use reported COVID-19 cases as a practical proxy for exposure and excess mortality as the outcome variable, acknowledging that both indicators are influenced by underreporting and measurement limitations.

A growing body of evidence suggests that socioeconomic and demographic structure, collectively termed National Framework Conditions (*NFCs*), were key drivers of this gap, particularly in middle-income countries [19–22]. Yet most studies assess these conditions using individual indicators, such as gross domestic product (GDP), prevalence of respiratory diseases, or healthcare spending without accounting for their interactions or structural configurations, such as the relationship between median population age and healthcare expansion [8, 23–27]. These univariate or additive models may obscure the systemic nature of pandemic vulnerability.

Understanding these systemic vulnerabilities is essential to examine how the risks countries face are structured through their socioeconomic and institutional conditions. The purpose of this analysis is to identify and characterize the coping capacity of countries within this structural context, positioning pandemic response as part of a broader system of national resilience. By linking these conditions to health outcomes, we aim to reveal the underlying configurations that shaped the pandemic’s unequal impact and to provide insights for strengthening resilience and reducing vulnerability to future global risks.

To overcome the limitations of these models, we propose a new approach. We jointly analyze a broad set of socioeconomic, demographic, and structural indicators from the WDI database comprising 1477 dimensions. Recognizing that development is nonlinear and context-dependent, we use a nonlinear dimensionality reduction method (e-Isomap) to project these variables into a low-dimensional latent space. This latent representation, which we call compressed National Framework Conditions (*cNFCs*), captures the essential variation in development structures while preserving their non-linear relationships [29, 30].

We use these *cNFCs* as input to a machine learning model that predicts excess mortality, alongside controls for pandemic exposure and known epidemiological risk factors. SHAP (SHapley Additive exPlanations) values allow us to quantify the relative contributions of socioeconomic structures, pandemic indicators (such as reported cases), and traditional health risk metrics to the model’s predictions of excess mortality. While the model is constrained by data availability and reflects associations rather than causal mechanisms, it provides a coherent and interpretable framework for assessing how national conditions shaped the global toll of the pandemic and may inform preparedness for future health crises.

## Methods

This study aims to identify how socioeconomic conditions contribute to the gap between reported COVID-19 case counts and observed excess mortality. To close this gap, we assemble data from three key domains: socioeconomic, pandemic indicators, and epidemiological features.

We begin by introducing excess mortality as our dependent variable, comparing estimates from multiple sources to justify our choice. Next, we present our socioeconomic data, drawn from the WDI, and describe our approach to compressing this high-dimensional dataset into a smaller set of interpretable components referred to as *cNFCs*. Based on previous work, these latent dimensions are characterized in terms of their thematic and structural properties [30].

In addition, we include a wide set of epidemiological features and pandemic indicators as control variables, including reported COVID-19 cases per million, Vaccinations per hundred, the Oxford Coronavirus Government Response Tracker stringency Index (Oxford stringency index), and other known risk factors (Table 1).

All components are integrated into a predictive machine learning model. This framework allows us to quantify the unique contribution of the *cNFCs* to excess mortality, while controlling for pandemic factors. We estimate the predictive influence of each variable using interpretable machine learning techniques (SHAP values). This SHAP values reveal which variables are important, providing insight into how structural and dynamic factors jointly shaped pandemic outcomes.

### Excess mortality data

We used the *P*-score of the excess mortality as our outcome variable, as defined by the WHO. The *P*-score is the percentage difference between observed and expected all-cause mortality. Importantly, it includes both direct deaths from COVID-19 and indirect deaths resulting from disruptions to healthcare systems, social services, and economic activity [1, 12].

As a health outcome metric, excess mortality is robust and less prone to biases than reported COVID-19 deaths, which can vary significantly across countries due to differences in case definitions, diagnostic capacity, reporting infrastructure, and cause-of-death attribution [13, 31–33].

Many excess mortality estimates have been published [1, 2, 7, 9]. The two most prominent, which we compared, show high similarities between the estimated values per country (Supplementary Material 1: Chapter S2 and Supplementary Material 1: Fig. S1). However, they differ in coverage and methods. We choose the WHO’s excess mortality estimates for 2020–2021 due to their broad global coverage (194 countries) and standardized methodology [1].

The WHO’s representation of excess mortality as a *P*-score (see Eq. 1) allows for meaningful cross-country comparisons by accounting for population size and baseline mortality rates.

1 $$\begin{aligned} P\text {-score}_{c,t} = 100 \cdot \frac{\text {Reported Mortality}_{c,t} - \text {Expected Mortality}_{c,t}}{\text {Expected Mortality}_{c,t}} \end{aligned}$$

In this representation, *c* denotes a country and *t* the year. We aggregated *P*-scores for 2020 and 2021 for all countries available in both the excess-mortality dataset and the WDI. Extreme outliers were defined as values with an interquartile range (IQR), the difference between the 75th and 25th percentiles, more than four times greater than the median ($\text {IQR}=12.42$, median = 8.96). Following this criterion, Peru’s data for 2020 (*P*-score = 91.09) and 2021 (*P*-score = 102.81) were identified as outliers and excluded from further analysis. The final sample comprised *P*-scores for 174 countries across 2 years ($n = 348$).

### World development indicators and *NFC*

The WDI comprises 1477 socioeconomic, environmental, and health-related indicators indicators from 217 countries (1960–2022) [34]. Many of these variables are highly correlated. For instance, “Access to electricity (% of population)” and “Life expectancy at birth, total (years)” are strongly correlated with $dcor = 0.8$. In fact, it has been shown for the WDI data earlier that these data are highly compressible with methods taking the non-linear structure into account [30]. The compressed data lie in a latent space which we will interpret as the *cNFCs*.

Linear methods, such as principal component analysis (PCA), do not capture the non-linear relationships present in socioeconomic data. To reduce the high dimensionality of the WDI dataset, we applied a non-linear dimensionality reduction technique (Supplement material chapter S2). We used Isomap, a technique that preserves geodesic distances between data points in a lower-dimensional embedding, thereby maintaining the global structure of the original data [29]. Isomap is sensitive to missing data, which is common in global development datasets. We therefore employed a robust variant known as Ensemble Isometric Feature Mapping (e-Isomap) [30]. This method generates multiple geodesic distance matrices from gap-filled subsets of the data and combines them into a stable embedding. This ensemble approach has previously been applied to an earlier version of the WDI. We successfully extended it to a more recent and expanded WDI dataset, enabling a stable and interpretable compression of 1477 socioeconomic indicators to a set of low-dimensional latent variables (*cNFCs*) (Supplementary Material 1: Fig. S3).

We limited the dataset’s temporal scope to 1990–2021 to maximize data quality and consistency. We also retained only indicators relative to population size (e.g., “Population, female (% of total population)” instead of “Population, female (total)”) and excluded indicators with more than 50% missing values. Despite maximizing data availability, 16% of values are missing. Only for the most developed countries is the data almost complete, while low- and lower-middle-income countries show a high proportion of missing data. We addressed this by limiting the temporal scope with minimum missing values and applying gap-filling procedures [30]. The final dataset before dimension reduction comprises 503 indicators from 180 countries (1998–2021).

### The *cNFC* dimensions

We compressed the WDI dataset into low-dimensional latent space using e-Isomap, which lowers the redundancy and reveals the main structural components of socioeconomic variation (Supplement material S3). This method preserves both linear and non-linear relationships and enables interpretable components, referred to as *cNFCs*, that can be traced back to the original indicators using the non-linear correlation method energy distance correlation (*dcor*) [35, 36].

To identify an appropriate dimensionality reduction approach, we compared several methods, including PCA and e-Isomap using different gap-filling strategies. Specifically, we examined how well the low-dimensional data representations could explain excess mortality across countries measured in terms of $R^2$ and residual mean squared error (RMSE). Excess mortality itself was not used to construct or tune the *cNFCs* themselves. We found that e-Isomap with ten dimensions provided the most stable and interpretable representation, and used it throughout the analysis (Supplement material S4).

The *cNFCs* reflect distinct aspects of national development. In our model we include the most recent representations of ten *cNFCs* numbered by their amount of explained variance (Supplement Fig. 2a). For reasons of interpretability we are focusing here on describing the ones that will have the highest importance for predicting excess mortality in the analysis later in the paper. The association with the indicators is traced back with energy distance correlation and visualized Supplementary Material 1: Fig. S3.

**Fig. 2:**
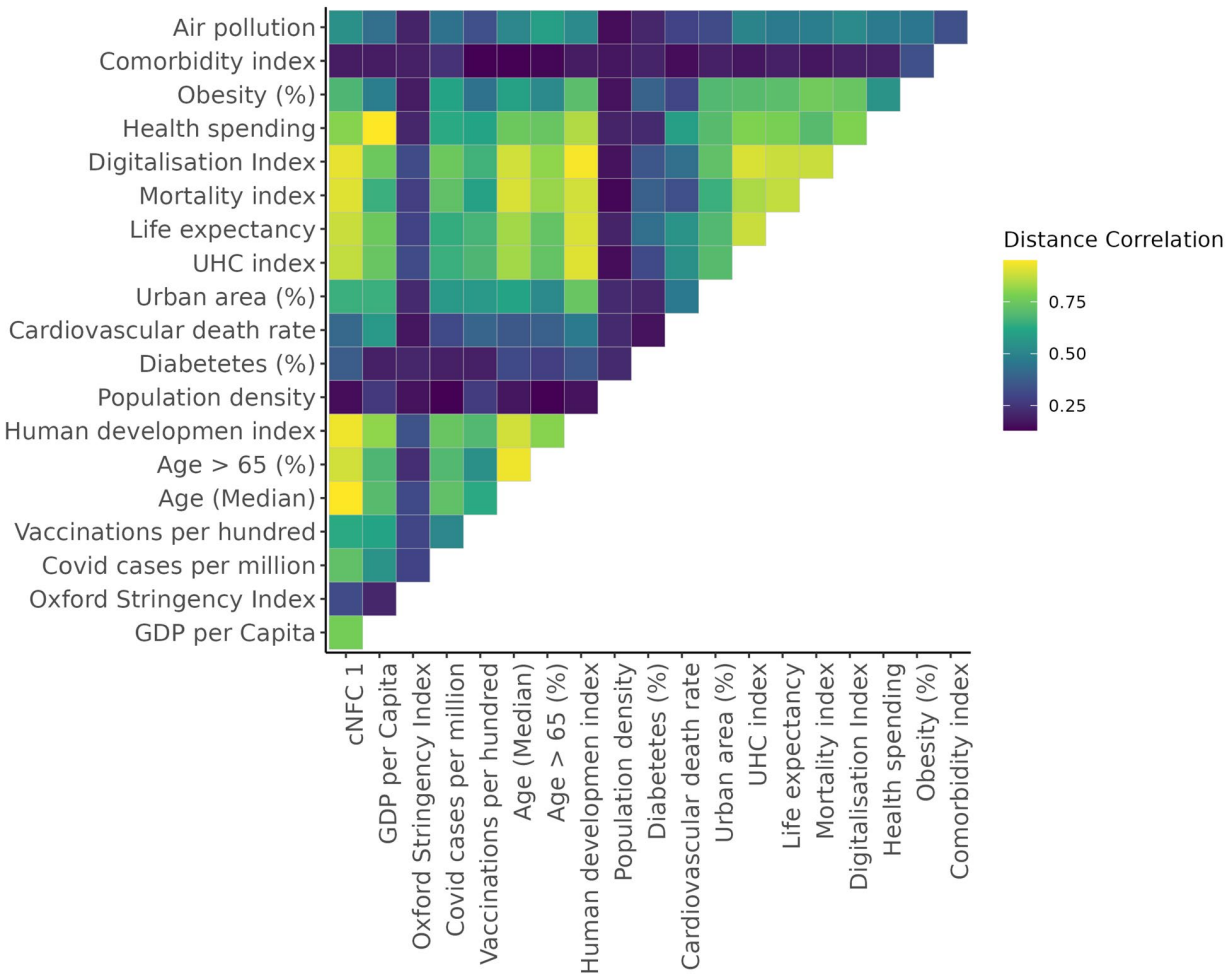
Heatmap with pairwise energy distance correlation. The map includes epidemiological features, pandemic preparedness indicators, and cNFC dimension 1 for 139 countries. UHC denotes to the Universal Health Coverage, GDP denotes gross domestic product and *cNFC 1* is the first dimension of the compressed National Framework Conditions

- *cNFC 1* represents 74% of the variance in the WDI dataset (Supplementary Material 1: Fig. S2a) and represents a classic economic-development gradient in which aging populations and declining fertility accompany a transition from agrarian to service-based economies. Consistent with findings by [30], it is strongly correlated with the Human Development Index, and many of the *epidemiological features*, as shown in Fig. 2. We find that *cNFC 1*’s middle-range value countries have the highest excess mortality, reflecting a similar non-linear relationship as the World Bank income classifications.
- *cNFC 2*, which explains 8% of variance in the WDI, reflects structural economic differences, particularly those associated with trade and labor force composition, and the energy intensity of the economy. High values in *cNFC 2* indicate economies driven by diversified service sectors. Lower values suggest reliance on primary sectors and manufacturing. *cNFC 2* shows countries on the lower end, which are characterized by primary sector and manufacturing dependence than the service-oriented economies.
- *cNFC 3* captures aspects of public sector employment and mortality rates, particularly in lower-middle-income countries. It is moderately correlated with “Death rate, crude (per 1000 people)” ($dcor = 0.69$) and “Ratio of female to male labor force participation rate (%)” ($dcor = 0.63$).
- *cNFC 4* covers aspects of economic profiles but is mostly a gradient between regions. High values are associated with the Gulf states such as United Arab Emirates and Kuwait, while low values such as Paraguay and Bolivia are in Latin America.
- *cNFC 8* is associated with indicators of public health vulnerabilities and economic reliance on tourism and external aid. High *cNFC 8* values are found in countries like Samoa, Vanuatu, and São Tomé and Príncipe. In contrast, low values in Brazil and Colombia. *cNFC 8* correlates with “International tourism, receipts (% of total exports)” ($dcor = 0.35$), “Incidence of HIV, ages 15–24 (per 1000 uninfected)” ($dcor = 0.35$), and “Trade in services (% of GDP)” ($dcor = 0.34$), linking this dimension to economies that are vulnerable to external public health challenges and service-based exports.

### Pandemic indicators and epidemiological features as covariates

The covariates are grouped into two categories based on their role in the analysis. They are collected from various sources and described in Table 1. *Pandemic indicators* reflect reported pandemic-related metrics directly linked to the pandemic experience. *Epidemiological features* are risk factors potentially associated with the excess mortality.

*Pandemic indicators* represent the key pandemic trajectories. We selected the Oxford Stringency Index, COVID-19 Cases per million, and Total vaccinations per hundred to incorporate government responses, infection prevalence, and population immunity [6, 37]. We also examined the cumulative infection–fatality ratio (IFR) from the IHME to account for differences in age structure and infection ascertainment across countries. After aggregating IFR estimates to annual values, its inclusion did not materially affect model performance or the ranking of the main feature groups, and IFR exhibited low predictive importance. The additional analysis is published in our Github repository.

**Table 1:** Pandemic indicators and epidemiological features considered in the analysis. Data sources include Our World in Data (OWID), State of Global Air report (SGA) and the World Bank World Development Indicators (WDI). HDI denotes the Human Development Index, GDP denotes gross domestic product, PPP purchasing power parity, UHC Universal Health Coverage, and PM$_{2.5}$ fine particulate matter with aerodynamic diameter below 2.5 μm

Variables	Description	Data source
**Pandemic indicators**		
Oxford stringency index	Oxford stringency index	OWID
COVID-19 cases	New reported COVID-19 cases per million	OWID
Vaccinations per hundred	COVID-19 vaccine doses administered per 100 people	OWID
**Epidemiological features**		
Life expectancy	Life expectancy	OWID
Age>65 (%)	Percentage of population older than 65	OWID
Obesity (%)	Prevalence of obesity	OWID
Age (Median)	Median age of population	OWID
Pop density	Population density	OWID
Diabetes (%)	Prevalence of diabetes	OWID
Cardio. death rate (%)	Cardiovascular death rate in percent	OWID
Urbanization	Percent of urban population	OWID
Handwashing facilities (%)	Percent of population with access to basic handwashing facilities	OWID
HDI	Human Development Index	OWID
GDP per Capita (PPP)	GDP per Capita adjusted by purchasing power parity	WDI
Air pollution	Yearly average PM$_{2.5}$ exposure	SGA
UHC index	Universal Health Coverage index	WDI
Health spending	Health expenditure per capita	WDI
Hospital beds	Hospital beds per thousand	OWID
Comorbidity index	Burden of disease indicators	OWID(1)
Digitalization index	Digitalization indicators	WDI(1)
Mortality index	Mortality of non-natural causes	WDI(1)
(1) compressed into a single indicator with PCA		

*Epidemiological features* set comprises established epidemiological risk factors such as obesity prevalence [38]. We also incorporated composed indices that might impact the pandemic resilience analog to previous publication [24]. One example Comorbidity Index, which is constructed out of the first principle component (93% explained variance) of the Global Burden of Disease dataset [39].

### Feature selection and data preparation

To identify independent features, we tested the correlation between all ten *cNFC* dimensions, the *Pandemic indicators*, and the *Epidemiological features*

We ensured that retained variables contributed distinct and independent information by calculating distance correlation between all features. We excluded 15 variables with a correlation higher than 0.75 and always the one with he lower coverage. We included as many features as possible while maintaining global coverage. For variables with less than 10% missing data, we applied probabilistic PCA for gap filling [40]. Variables with insufficient observations across countries, such as Handwashing Facilities, Cardiovascular Death Rate, and Air Pollution, were excluded from the analysis.

The final feature set used in the model includes 18 variables: ten *cNFC* dimensions (ordered by the share of WDI variance they explain), three *Pandemic indicators* (COVID Cases per million, Oxford Stringency Index, and Vaccinations), and five *Epidemiological features* (Population Density, Urban Area in %, Diabetes Prevalence, Obesity Prevalence, and the Comorbidity Index).

The ten *cNFC* dimensions were selected using the most recent available values preceding the corresponding *P*-score for each country-year pair. Accordingly, excess mortality in 2020 was modeled with *cNFC* values from 2019, and mortality in 2021 with values from 2020. This 1-year lag ensures that a country’s socioeconomic and institutional conditions reflect the situation prior to the pandemic outcomes, avoiding circularity in the analysis.

Our analysis is structured around the three feature domains: *pandemic indicators*, *epidemiological features*, and the *cNFCs*. To ensure independence among variables, we used pairwise distance correlation as a filter. Features with $dcor>0.75$ with any *cNFC* were removed to avoid collinearity. In total, eight epidemiological features were excluded in this step. Notably, median age ($dcor = 0.94$), the Human Development Index ($dcor = 0.93$), and digitalization ($dcor = 0.92$) were eliminated due to high correlation with *cNFC 1* (Fig. 2). This *integrated model* included 18 features: 10 *cNFCs*, 3 pandemic indicators, and 5 epidemiological controls.

### Evaluating the model and feature importance SHAP values

**Fig. 3:**
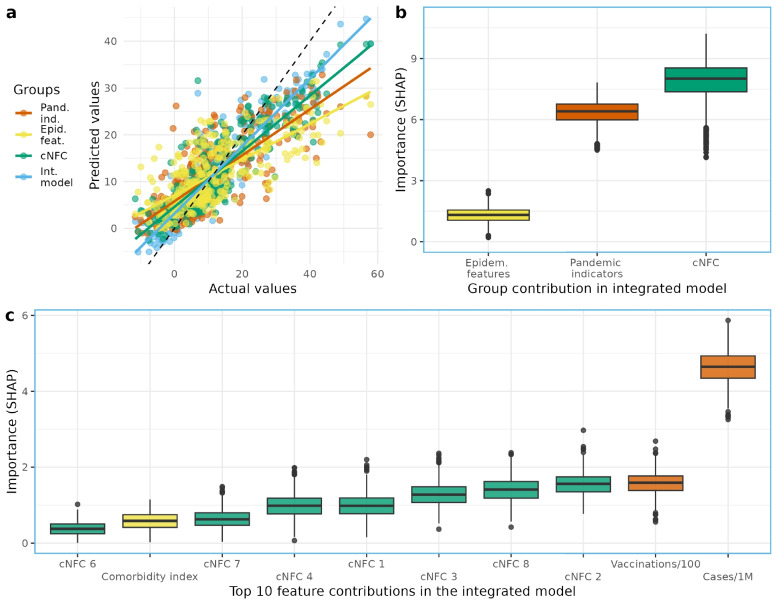
Contributions of different components of variables to the prediction of excess mortality. **a** Model performance for each component separately (Pand.ind. = pandemic indicators; Epid.feat. = epidemiological features; cNFC = compressed National Framework Conditions) and combined in the integrated model (Int. model). The relationship between estimated and observed excess mortality is illustrated by the scatter points, with the dashed line indicating the 1:1 reference. Performance differences across groups are visualized through the deviation from the reference line and the slope of the group-specific regression fits. **b** Contribution of all three model components included into the integrated model. **c** Contribution of each features to the overall performance of the integrated model

**Fig. 4:**
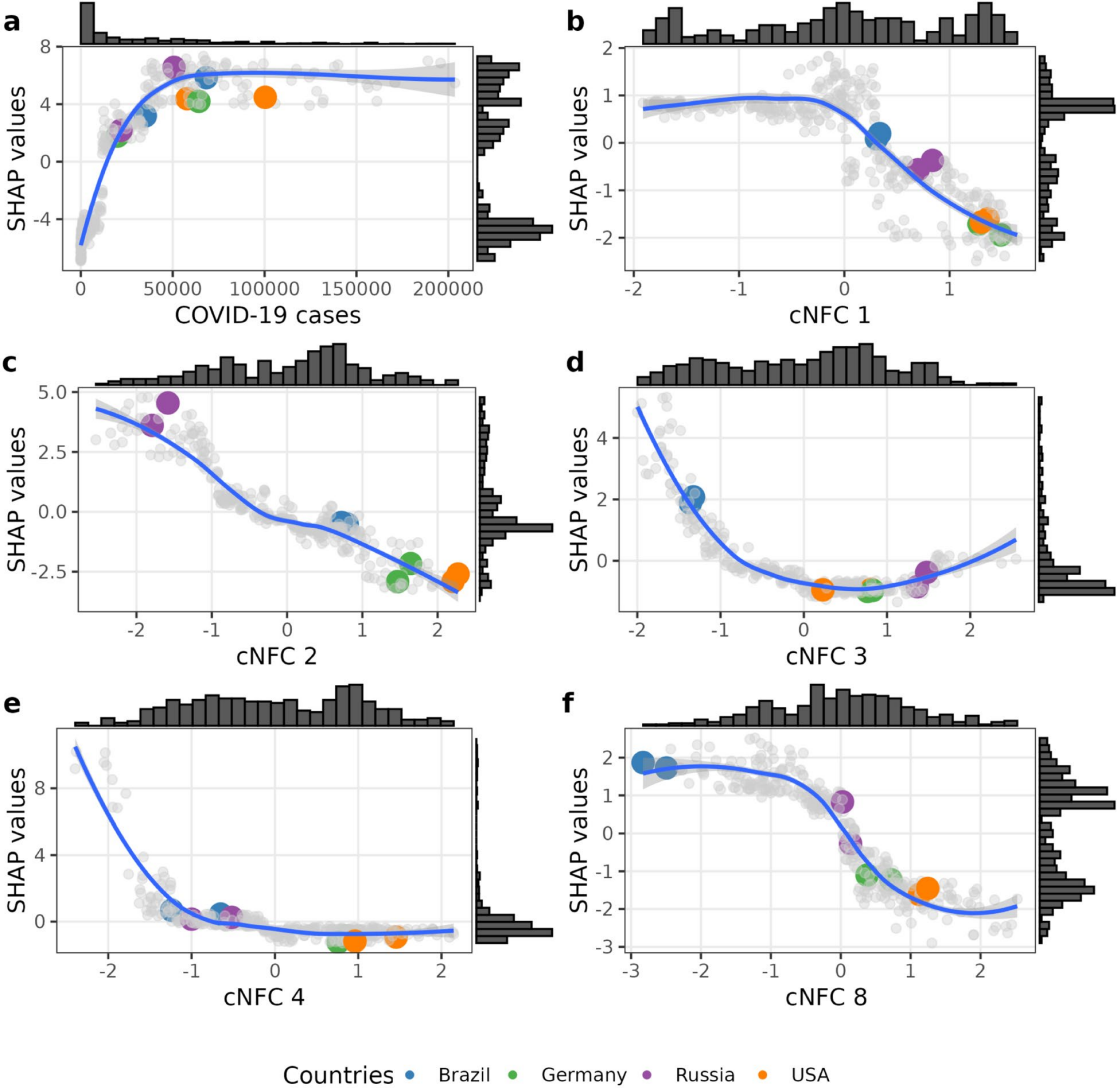
Variable contributions to the estimated excess mortality. The six panels display different features of the integrated model on the *x*-axis and their marginal contributions to excess mortality on the *y*-axis, quantified using SHapley Additive exPlanations (SHAP) values. Example countries are highlighted for illustration. Each panel also includes a histogram showing the distribution of the two annual observations per country ($n = 348$). The term *cNFC* refers to compressed National Framework Conditions

We used SHAP values to measure the feature importance. SHAP values are based on a concept from game theory and assign each feature a value that reflects its contribution to a specific prediction [41]. SHAP values decompose the model prediction into additive components reflecting the marginal contribution of each feature. The SHAP values show how much each feature increased or decreased the predicted excess mortality. To provide a robust measure feature importance, we used the median absolute SHAP from all runs. SHAP is a well established explainable AI method to quantify marginal contributions of features and the general feature importance [42–45]. Visual outputs of this analysis are shown in Figs. 3 and 4.

### Model training and validation

Our model is tailored around the trade-off between exploring the interactive effects of the features and avoiding overt-fitting. We chose a low learning rate ($\eta =$0.1) and a low number of rounds ($\text {rounds}=100$), and restricted the maximum depth of the trees to three. We evaluated the predictive performance of model components with a tree-based regression model: eXtreme Gradient Boosting (*XGBoost*) in R [46]. The mean annual excess mortality data was split into training (80%) and test (20%) sets, ensuring that each country was only present in either the train or test set. To avoid over-fitting, we used 10-fold cross-validation and adjusted the hyper-parameter.

We reran the prediction 1000 times with different sub-samples of the data to increase its robustness. Model performance was evaluated using the coefficient of determination ($R^2$), commonly employed in similar research, and RMSE. Due to strong variation between each run, we report the median and IQR as summary statistics.

### Software and reproducibility

All analyses were conducted in R (v4.2.1) using *xgboost*, *SHAPforxgboost*, and custom code based on Kraemer et al. [30]. The code is available on GitHub [47] together with a link to preprocessed *cNFC* correlation table.

## Results

### Global predictive model of COVID-19 excess mortality

We introduce the first global-scale model of COVID-19 excess mortality covering a wide range of socioeconomic conditions (*cNFCs*). Our machine learning model explains nearly half of the observed variance in excess mortality ($R^2$: median 49.7, IQR: 10.9; Fig. 3a, Supplement S7 and Supplement Fig. S5).

To evaluate model performance, we compared predictions from the integrated *cNFC* model with those derived from its individual components: *cNFCs*, *pandemic indicators*, and *epidemiological features*. Each component in isolation explains approximately 30–35% of the variance in excess mortality (Supplementary Material 1: Table S1). In contrast, the integrated *cNFC* model yields markedly superior predictive accuracy. In 1000 runs, the integrated model achieves a higher explained variance ($R^{2}$) and lower prediction error (Supplementary Material 1: Fig. S5). Figure 3a illustrates that the predicted excess mortality values are substantially closer to the observed values, demonstrating an overall improvement. Collectively, these results indicate that integrating all feature groups provides a more accurate and robust representation of excess mortality patterns than any individual component alone.

We quantify the importance of each feature using SHAP values, a widely used explainable AI method for assessing feature contributions in machine learning models [42, 43, 45]. To ensure robustness, we rely on absolute SHAP values, which provide a stable measure of importance across repeated model runs. As a reference point, the sum of SHAP values across all features remained largely consistent across runs (median: 15.7, IQR: 1.8). We therefore interpret the SHAP values of individual feature groups as their proportional share of this total contribution.

The *cNFC* feature set had the strongest influence (median: 8.1, IQR: 1.2), followed by *pandemic indicators* (Median: 6.4, IQR: 0.7) and, far behind, the *epidemiological features* (median: 1.3, IQR: 0.3; Fig. 3b). This highlights the central role of structural socioeconomic conditions in explaining pandemic-related mortality.

We next examined individual features to evaluate the contribution to the overall performance in a *integrated model* (Fig. 3c). This *integrated model* combines the features of all three components as defined in the Methods section. COVID-19 cases contributed the most to the predicted outcome (median: 4.4, IQR: 0.6), followed by *Vaccination* (median: 1.6, IQR: 2.2), *cNFC 2* (median: 1.5, IQR: 0.4), *cNFC 8* (median: 1.4, IQR: 0.4), *cNFC 3* (median: 1.3, IQR: 0.4), *cNFC 1* (median: 1, IQR: 0.3), and *cNFC 4* (median: 1, IQR: 0.4). COVID-19 Cases make the strongest contribution to the predicted excess mortality. Vaccination and *cNFC 2* are similar, closely followed by the other *cNFCs*.

These findings suggest that *cNFCs* effectively capture the structural information embedded in conventional health and demographic indicators. By contrast, the contribution of the *epidemiological features* was minimal, with only the Comorbidity Index ranking within the 10 most influential features, eighth overall (Fig. 3c). As shown in the feature selection process (Fig. 2), eight out of 18 epidemiological features were removed prior to modeling due to high correlations with *cNFC 1*. Notably, median age ($dcor = 0.94$), the Human Development Index ($dcor = 0.93$), and digitalization ($dcor = 0.92$) exhibited particularly strong associations. These results indicate that, once *cNFCs* are included, many traditional health risk indicators contribute little additional predictive value.

### Socioeconomic and demographic drivers of pandemic outcomes

Building on the model performance results, we now explore how individual *cNFC* components shape cross-country risk profiles. Despite their non-linear structure, the contributions of *cNFCs* can be traced back to underlying socioeconomic indicators (WDI), as described in the Methods and in the Supplementary Material 1: Chapter S5. The *cNFCs* summarize distinct dimensions of national development: *cNFC 1* reflects overall development and demographic transition, *cNFC 2* economic structure and labor-force composition (with pronounced differences in the age distribution of the working population), *cNFC 3* labor and institutional stability, *cNFC 4* regional economic orientation, and *cNFC 8* public health vulnerability and external dependence. Corresponding indicator correlations are described in the “Methods” section and Supplementary Material 1: Fig. S3. Using relative SHAP values allows us to identify how specific *cNFCs* interact with the development context of each country and how they contribute directionally to excess mortality (Fig. 4).

In Fig. 4a, we show the relationship between COVID-19 cases per million and their SHAP contributions. The histogram along the *x*-axis shows a right-skewed distribution of case numbers, with most countries reporting relatively low case rates. However, the SHAP values plateau, indicating that higher reported case numbers do not translate into proportionally higher predicted excess mortality.

Figure 4b reveals a threshold effect for *cNFC 1*, which encodes a gradient of general development. At lower values characteristic of less developed countries SHAP values are positive, contributing to higher excess mortality. As values increase, SHAP contributions turn negative, suggesting that more developed countries are associated with mortality mitigation.

Figure 4c illustrates a near-linear inverse relationship between *cNFC 2* and excess mortality. Countries with low *cNFC 2* values show strong positive SHAP contributions, reflecting heightened vulnerability. Figure 4d and e show that *cNFC 3* and *cNFC 4* exert influence only at the lower end of their value range, affecting a small subset of countries. Figure 4f shows a bimodal SHAP distribution for *cNFC 8*, suggesting that its contribution is highly context-specific and likely binary in nature.

###  Country-level risk profiles

To illustrate how *cNFC*-based patterns manifest in concrete national contexts, we examine four example countries: Germany, Russia, the USA, and Brazil. Their respective positions within the *cNFC* space provide insight into underlying socioeconomic risk structures (Fig. 4). Looking into the underlying WDIs, we trace key indicators strongly associated with each *cNFC* dimension and compare representative indicators across the four countries. Table 2 presents a selection of these indicators, chosen for their strong and consistent association with specific *cNFCs*. Each variable exemplifies broader patterns embedded in the respective dimensions. These comparisons highlight the distinct combinations of socioeconomic characteristics. A more detailed view of the thematic coverage for each component is available in Supplementary Material 1: Fig. S3.

**Table 2:** Comparison of selected WDI for Germany (DEU), Russia (RUS), the United States (USA), and Brazil (BRA)

Indicator	Max cor	DEU	RUS	USA	BRA
Life expectancy (years)	*cNFC 1*	79.99 (1.96)	68.84 (5.65)	78.39 (1.15)	73.18 (2.55)
GDP per capita ppp(log USD)	*cNFC 1*	4.68 (0.06)	4.40 (0.10)	4.74 (0.04)	4.15 (0.07)
Youth labor force rate (%)	*cNFC 2*	50.42 (1.57)	39.38 (2.9)	50.81 (5.23)	59.36 (5.84)
Health spending (log USD)	*cNFC 2*	3.67 (0.13)	2.71 (0.28)	3.89 (0.15)	2.86 (0.30)
CO_2_ damage (% of GNI)	*cNFC 2*	0.66 (0.09)	4.04 (1.71)	0.99 (0.09)	0.89 (0.33)
Death rate (per 1,000)	*cNFC 3*	10.5 (0.9)	14.2 (2.3)	8.3 (0.4)	6.31 (0.23)
Female labor force (%)	*cNFC 3*	45.95 (1.11)	48.81 (0.55)	46.05 (0.16)	42.6 (0.59)
Homicides (per 100k)	*cNFC 4*	0.99 (0.38)	11.65 (13.64)	5.12 (0.83)	26.63 (2.16)
Tourism receipts (% exports)	*cNFC 8*	3.32 (0.45)	3.43 (0.63)	9.21 (1.15)	2.8 (0.71)
HIV incidence (per 1000)	*cNFC 8*	0.03 (0.01)	0.68^1^ (0.19)	0.12 (0.01)	0.25 (0.01)

Median and interquartile range (IQR) values are reported for each indicator from the World Development Indicators (WDI) over the period 2010–2020. Country codes denote Germany (DEU), Russia (RUS), the United States (USA), and Brazil (BRA). The column “Max cor” indicates the compressed National Framework Condition (cNFC) dimension most strongly associated with each indicator. Indicator labels are abbreviated for readability. Gross domestic product (GDP) per capita (purchasing power parity, PPP) and health spending values are log$_{10}$-transformed and reported in US dollars (USD). CO$_2$denotes carbon dioxide, and GNI denotes gross national income. HIV denotes human immunodeficiency virus. ^1^HIV incidence data for Russia are missing in the WDI; for visualization, values were taken from published Global burden of disease data [48]

Russia and Germany reported similar case numbers during 2020–2021 (Russia: 21.8k and 50.7k; Germany: 19.9k and 64.2k per million), yet experienced starkly different mortality outcomes. Russia’s excess mortality reached 20.9% (2020) and 46.4% (2021), compared to Germany’s 7.3% and 14.4%. Both countries scored similarly on *cNFC 1, 3, 4*, and *8*, dimensions generally associated with more developed health systems. Indicators like Life expectancy and GDP per Capita ppp are positively associated with *cNFC 1*, reflecting similarities in the development state. However, the countries diverge sharply on *cNFC 2*, which represents structural economic features and Labor force structure and demographics (Supplementary Material 1: Fig. S3). Russia scores lower, corresponding with high positive SHAP values. Underlying indicators include CO_2_ damage (% of GNI)(Russia: 4.04; Germany: 0.89) and Youth Labor force rate (Russia: 39.38%, Germany: 50.42%), highlighting Russia’s structural vulnerability.

Brazil and the USA provide another instructive pair. Although Brazil reported fewer cases (35.1k and 68.2k) than the USA (57.8k and 100.3k), its excess mortality in 2021 was more than double (33.1% vs. 15.0%). The USA’s high values on *cNFC 1* and *cNFC 2* correspond with lower SHAP values, suggesting resilience. In contrast, Brazil’s elevated mortality is associated with high SHAP contributions from *cNFC 3* and *cNFC 8*, which reflect regionally specific vulnerabilities, particularly in Latin America.

These case comparisons highlight a key insight: while reported pandemic indicators provide part of the picture, structural factors encoded in the *cNFCs* are essential to understanding excess mortality. SHAP based interpretation allows us to pinpoint which latent dimensions shape each country’s risk profile, making the model not only predictive but interpretable.

## Discussion

Our model shows that *cNFCs* explain more variation in excess mortality during the COVID-19 pandemic (2020–2021) than reported infections, vaccination rates, or classical epidemiological risk factors. With the *cNFCs* this approach offers a new perspective on structural pandemic vulnerability, particularly illuminating why upper-middle-income countries experienced disproportionately high mortality despite fewer reported cases.

Existing literature underscores that socioeconomic conditions are central determinants of COVID-19 health outcomes, not merely background variables. A large body of work links social and economic inequalities to systematic differences in morbidity and mortality, partly via unequal access to healthcare, differences in occupational exposure, and broader disparities in housing, income security, and living conditions [20, 49]. Macroeconomic and demographic characteristics have further been associated with the severity of pandemic impacts across regions, indicating that structural vulnerabilities shape both patterns of transmission and the capacity of health systems to absorb shocks [22, 27, 50].

However, in the literature the socioeconomic factors are usually studied as isolated, additive variables, which fails to capture their interdependence [9, 24, 25]. The World Bank’s WDI database alone includes nearly 1500 variables. Prior research has shown that socioeconomic indicators lie on a lower-dimensional manifold [30]. By applying non-linear dimensionality reduction, we derived *cNFCs* that represent these latent structures of development. This supports a shift in focus from individual vulnerabilities to systemic development configurations.

Our results show that *cNFC 1* captures much of the same variance as classical indicators like GDP per capita, life expectancy, and HDI but with stronger predictive power for pandemic mortality. This suggests that vulnerability is shaped by a broader non-linear structural context rather than by individual indicators in isolation. Modeling development through integrated components like *cNFC 1* avoids overlap and redundancy among socioeconomic and demographic variables and helps explain why several middle-income countries experienced disproportionately high excess mortality despite fewer reported infections.

*cNFC 2* emerged as nearly as predictive as vaccination coverage. It reflects economic structure, particularly reliance on manufacturing and primary sectors, and shows regional clustering in countries with post-Soviet or state-controlled economic legacies. These findings align with earlier work highlighting how historical development paths shape pandemic response capacities [51]. Negative values of *cNFC 2*, which cluster among former Soviet states, are associated with high Shapley values, indicating strong relevance in mortality prediction. This suggests that historical economic legacies, such as those of the Cold War, play a measurable role in pandemic outcomes.

These insights suggest that pandemic vulnerability is systemic and structurally embedded. Our findings emphasize that national resilience is not determined solely by pandemic policies or immediate health capacities but also by broader, historically shaped development conditions. By grouping structurally similar countries through *cNFCs*, we offer a framework for identifying where and why countries experience elevated risk.

Previous work has shown that *cNFCs* exhibit regional clustering [30]. This broader structural organization is consistent with the patterns identified in our analysis and is further illustrated in Supplementary Material 1: Chapter S6. The rerun of these representation The *cNFCs* reveal that countries with shared historical and geographical backgrounds occupy similar positions in the latent space, forming distinct developmental configurations. Because these patterns representing combinations of socioeconomic and institutional characteristics, the model’s predictive power depends on which parts of this structural space are included in training. Randomly excluding countries removes portions of this space and changes the systemic context from which predictions are derived. The resulting variation between runs therefore reflects the uneven global distribution of risk and resilience rather than model instability.

Our study has several limitations. First, the WDI dataset offers only annual data, limiting our ability to track dynamic changes such as shifting government responses or virus waves. This temporal aggregation reduces the total number of observations and may lower the sensitivity of features that vary on shorter time scales. For example, the Oxford Stringency Index captures highly granular shifts in intervention intensity that closely follow wave- and country-specific exposure patterns. When aggregated to the annual level, its contribution to the excess mortality becomes weak (SHAP: median 0.14, IQR 0.15). A related limitation is that the Vaccinations per hundred data were also aggregated annually, even though vaccine availability was minimal in 2020 and increased earlier in upper-middle and high-income countries during [6, 52]. Second, while XGBoost captures non-linear interactions, it trades off interpretability. Though we mitigate this with SHAP values and dimensionality reduction, non-linear models remain less transparent than linear ones. Third, while we used WHO excess mortality estimates for their consistency and global coverage, different estimation methods yield varying results [1–3, 7]. We addressed this by validating our outcome data against Institute for Health Metrics and Evaluation Excess mortality estimates ($dcor = 0.867$) [6]. A fourth limitation concerns the cross-country comparability of reported COVID-19 case counts. Such indicators depend strongly on differences in testing capacity and infection ascertainment, i.e., the proportion of infections that are detected through testing and reporting infrastructure, across countries, which may bias international comparisons [53, 54]. Although adjusting for under-ascertainment would be desirable, consistent global estimates are not currently available.

Another challenge lies in interpreting the *cNFCs* themselves. These components are non-linear and cannot be fully mapped onto traditional variables. While we used distance correlation and regional context to guide interpretation, some components, particularly beyond *cNFC 2*, appear to encode geographic or historical clusters that are less directly policy-relevant. This makes the framework most suitable for identifying broad structural risk patterns rather than fine-grained national comparisons.

Despite these limitations, our framework provides an empirical basis for understanding structural vulnerability in global health. The finding (Fig. 3b) demonstrate the need to treat socioeconomic resilience as a pillar of pandemic preparedness. Future research can build on this by integrating time-varying data, exploring country clusters, or modeling intervention effectiveness across different structural profiles.

For policymakers, these findings highlight the importance of dual-track preparedness. Structural investments such as healthcare infrastructure, labor protections, and digital systems must complement rapid-response capabilities like vaccination campaigns. The limited success of existing preparedness indices [55, 56] calls for frameworks that reflect underlying structural resilience, not just health sector inputs.

These structural interventions should be tailored to a country’s position in the *cNFC* space. Preparedness efforts that ignore structural variation may overlook key vulnerabilities. We recommend that global health governance shift toward supporting systemic development in vulnerable middle-income countries, including technical support, economic diversification, and targeted healthcare investment.

## Conclusions

Our analysis shows that pandemic outcomes are shaped not only by exposure or response but also by deeply embedded structural socioeconomic conditions. Some of these arise from well-functioning welfare states, while others are rooted in the historical context of specific regions, such as the post-Soviet states. Both types of factors shape national vulnerability and must be addressed in future pandemic preparedness.

Our findings demonstrate that socioeconomic conditions are more than the sum of their parts. The strong correlation of most known risk factors with the first *cNFC* underscores the need to account for their interconnectedness. By reframing pandemic risk as a function of non-linear socioeconomic development, our approach provides an integrated perspective that goes beyond conventional indicators. The resulting framework offers an interpretable, globally applicable view of resilience and vulnerability from a single data source.

Looking ahead, future work must focus on translating these insights into actionable risk assessment tools. This requires advancing our theoretical understanding of the resources that explain the vulnerability of middle-income countries in particular and tailoring a framework that enables policymakers to identify and address the structural factors that make countries more at risk.

## Supplementary Information

Supplementary material 1. Supplementary Methods, Tables, and Figures. This file contains additional analyses and supporting material referenced in the main text. It includes Chapter S1 (Indicators of the pandemic health outcome), which provides correlation analyses between reported COVID-19 cases, deaths, and excess mortality estimates. Chapter S2 (Isomap dimensionality reduction) describes the Isomap algorithm and its application to the WDI dataset. Chapter S3 (Properties of the latent space representation of the WDI) presents comparisons between PCA and Isomap components and illustrates the non-linear structure of development. Chapter S4 (Benchmark performance of different feature sets) reports predictive performance across pandemic indicators, epidemiological features, and WDI-derived feature sets (Table S1). Chapter S5 (Tracing back the indicators: cNFC components and indicator structure) provides a mapping between WDI indicators and cNFC components (Fig. S1). Chapter S6 (Geographical patterns and shared development histories) illustrates regional clustering in the latent socioeconomic space (Fig. S2). Chapter S7 (Predictive performance of the integrated model) reports robustness analyses across 1,000 model runs and includes performance distributions (Fig. S3).

## Data Availability

All data used in this study are publicly available. WDI data were downloaded on 11 November 2023 from the World Bank database (https://datatopics.worldbank.org/world-development-indicators/). Covariates not included in WDI were obtained on 3 November 2023 from Our World in Data, specifically the Coronavirus Dataset (https://ourworldindata.org/) and the Burden of Disease Dataset (https://ourworldindata.org/burden-of-disease). Excess mortality data were obtained from the World Health Organization, available since 14 December 2022 as part of the publication by Msemburi et al. (https://doi.org/10.1038/s41586-022-05522-2). All data processing scripts are available on Github (https://github.com/knenovsky/Mortality-risk-of-COVID-19-pandemic-and-human-development.git).

## Abbreviations

AI: Artificial intelligence
BRA: Brazil
cNFC: Compressed National Framework Conditions
DEU: Germany
GDP: Gross domestic product
HDI: Human Development Index
HI: High income
IQR: Interquartile range
LI: Low income
LMI: Lower-middle income
NFC: National Framework Conditions
NPI: Non-pharmaceutical interventions
PCA: Principal component analysis
RMSE: Root mean square error
SHAP: SHapley Additive exPlanations
UHC: Universal Health Coverage
UMI: Upper-middle income
USA: United States of America
WDI: World Development Indicators
WHO: World Health Organization
XAI: Explainable artificial intelligence
