# Supplementary material for: Mortality risk during the COVID-19 pandemic is shaped by human development
Source: BMC Glob Public Health. 2026 Mar 2;4:23. doi: 10.1186/s44263-026-00255-0 (PMC12952034; doi:10.1186/s44263-026-00255-0)
Supplement: Supplementary file 1 — Supplementary material 1. Supplementary Methods, Tables, and Figures. This file contains additional analyses and supporting material referenced in the main text. It includes Chapter S1 (Indicators of the pandemic health outcome), which provides correlation analyses between reported COVID-19 cases, deaths, and excess mortality estimates. Chapter S2 (Isomap dimensionality reduction) describes the Isomap algorithm and its application to the WDI dataset. Chapter S3 (Properties of the latent space representation of the WDI) presents comparisons between PCA and Isomap components and illustrates the non-linear structure of development. Chapter S4 (Benchmark performance of different feature sets) reports predictive performance across pandemic indicators, epidemiological features, and WDI-derived feature sets (Table S1). Chapter S5 (Tracing back the indicators: cNFC components and indicator structure) provides a mapping between WDI indicators and cNFC components (Fig. S1). Chapter S6 (Geographical patterns and shared development histories) illustrates regional clustering in the latent socioeconomic space (Fig. S2). Chapter S7 (Predictive performance of the integrated model) reports robustness analyses across 1,000 model runs and includes performance distributions (Fig. S3). [file 44263_2026_255_MOESM1_ESM.pdf]

Supplementary material 1:

S1: Indicators of the pandemic health outcome

In our analysis we use COVID-19 Cases as a proxy for the real world pandemic experience and excess mortality, estimated by the WHO, to measure the overall health outcome. To illustrate our decision we show the relationship between reported COVID-19 Cases, COVID-19 Deaths and excess mortality. We align data from two authoritative sources: the "Our World in Data" (OWID) COVID-19 Dataset and the World Health Organization's (WHO) excess mortality estimations. Our analysis reveals a strong correlation between OWID reported COVID-19 cases and deaths figure S1 A). Furthermore, excess mortality estimations from both OWID and WHO demonstrate comparably low correlation with with reported COVID-19 cases (Figure S1 B,C). Notably, the excess mortality figures from both sources show strong alignment across most countries, suggesting consistency in these independent estimates (Figure S1 D).

S2: Isomap Dimensionality Reduction

Isomap is a non-linear dimensionality reduction method that preserves the intrinsic geometry of the data by maintaining geodesic (manifold-based) distances between data points. The algorithm proceeds in three main steps:

- **Constructing the Neighborhood Graph:** For each data point, identify its nearest neighbors based on Euclidean distance. These connections form a neighborhood graph where each point is linked to its closest neighbors.
- **Computing Geodesic Distances:** Estimate the shortest path (geodesic) distances between all pairs of points within the graph, typically using Dijkstra's algorithm.
- **Applying Multidimensional Scaling (MDS):** Perform classical MDS on the geodesic distance matrix to obtain a lower-dimensional embedding that preserves these distances as closely as possible.

By focusing on geodesic rather than linear distances, Isomap captures the nonlinear structure of the data. This is particularly useful when the data lies on a curved manifold, as it reveals latent structure more effectively than linear methods like PCA. A detailed description of the original Isomap algorithm is provided in [1], and its ensemble extension e-Isomap has been demonstrated in the context of socioeconomic indicators from the WDI dataset in [2].

S3: Properties of the Latent Space Representation of the WDI

A large amount of WDI is highly linear and can be represented by the first component of Isomap. The first component of PCA and Isomap are very similar (Fig. S2 Panel B). The WDI includes many indicators that can be linearized by the e-Isomap approach. The Example Fig. S2 Panel C and D illustrated the non-linear structure of development. This dimension reduction is similar to the results from previous work.

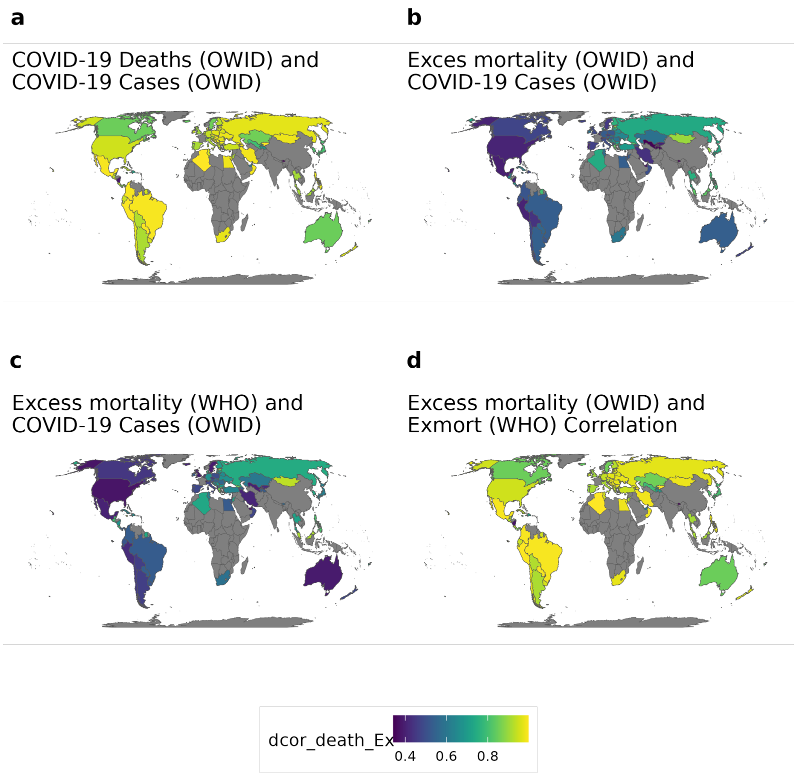

Fig. S1 Correlation analysis between key COVID-19 indicators. Energy distance correlations between (A) officially reported COVID-19 cases and OWID officially reported COVID-19 deaths, (B) OWID excess mortality estimates and reported COVID-19 Cases, (C) WHO excess mortality estimates and reported COVID-19 Cases and (D) excess mortality of both sources. The strong correlation between reported cases and deaths is evident, while both excess mortality estimates (OWID and WHO) show similar correlation patterns with reported cases. The high correlation between OWID and WHO excess mortality estimates across most countries indicates robust agreement between these independent data sources.

S4: Benchmark performance of different feature sets

We evaluated the predictive performance of different feature sets independently. Each feature set explains roughly one-third of the variance in excess mortality (Supplement Table). Models were tested in 1,000 runs with varying training and test splits to ensure robustness. We additionally compared different dimensionality reduction methods and numbers of representative features. Among these, the *cNFCs* achieved the best performance, combining high predictive accuracy with interpretability. Details on the compression process are provided in the Methods section.

To reduce redundancy and uncover interpretable patterns in the WDI dataset, we applied dimensionality reduction techniques. We benchmarked Principal Component Analysis (PCA), Isomap, and extended Isomap (e-Isomap), using both complete-case and imputed WDI datasets. For each method, we tested multiple dimensionalities,

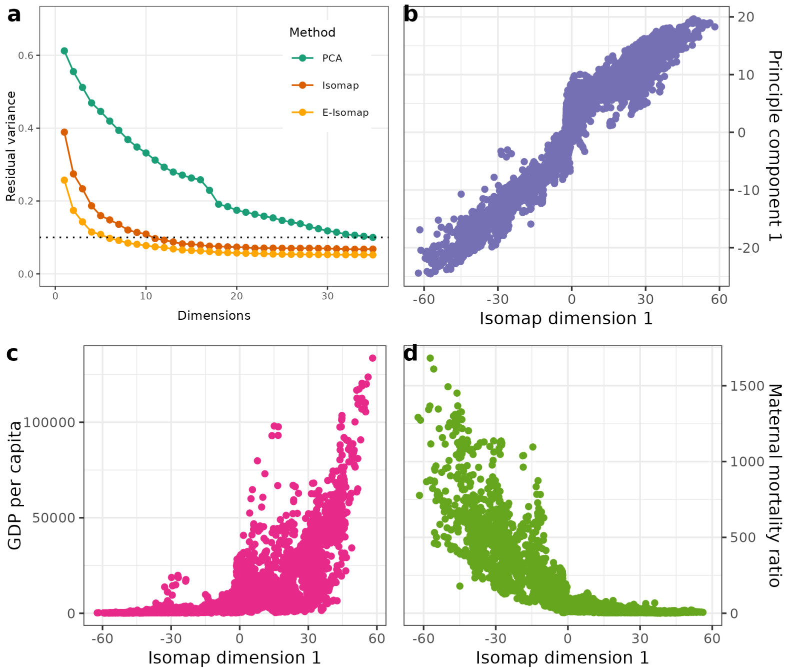

Fig. S2 Dimension reduction of the WDI. **Panel A:** The x-axis illustrates the number of dimensions resulting from dimension reduction, accompanied by their respective explained variance. The y-axis portrays residual, unexplained variance, with a 10% residual variance marked by a horizontal line. **Panel B:** Presents plots of Isomap 1 and Principal Component 1. **Panels C and D:** Depict Isomap dimensions against example indicators from the WDI. In Panel C, GDP per capita is visualised, and in Panel D, Maternal Mortality (per 100k live births) is displayed.

with performance assessed across 1,000 cross-validated runs. Each run used 20% held-out test data, and we recorded both explained variance (R^2) and prediction error (RMSE).

Among the approaches tested, e-Isomap consistently achieved the highest R^2 and lowest RMSE, demonstrating its ability to capture the non-linear structure of socioeconomic variation in the WDI dataset. This result supports its use in deriving the *cNFCs* applied in the main analysis.

S5: Tracing back the indicators: cNFC Components and Indicator Structure

The figure(Supplement Fig. S3) summaries the relationship between WDI and their associated *cNFC* components.

- **Dendrogram:** Organizes indicators thematically following the World Bank classification.
- **Colored lines:** Indicate the energy distance correlation between each indicator and its most associated *cNFC*, with *cNFC1* showing widespread associations across indicators.

Dataset	Features (N)	Explained Variance (R^2)	Prediction Error (RMSE)	Interpretability
**I. Pandemic indicators**				
Pandemic indicators	3	33.5% (27.3; 39.8)	0.88 (0.80; 0.95)	High
**II. Epidemiological features**				
Literature-based risk factors	19	31.0% (23.1; 39.4)	0.89 (0.80; 0.94)	High
**III. World Development Indicators**				
$\text{WDI}_{\text{Baseline}}$	149	24.7% (18.0; 31.0)	0.88 (0.80; 0.95)	Low
$\text{WDI}_{\text{Imputed}}$	503	32.4% (20.4; 38.4)	0.83 (0.76; 0.90)	Low
PCA	20	26.6% (20.1; 32.9)	0.87 (0.80; 0.93)	High
Isomap	10	24.5% (17.8; 30.8)	0.87 (0.81; 0.94)	Medium
e-Isomap	10	32.9% (26.5; 38.8)	0.83 (0.77; 0.89)	Medium

Table S1 Predictive performance of feature sets used to estimate excess mortality. WDI feature sets are limited to the 503 indicators included in dimensionality reduction. The baseline set includes only complete cases (2019–2020), while the imputed set incorporates missing data. Each configuration was evaluated over 1,000 runs with 20% held-out test data. Values for explained variance (R^2) and RMSE are reported as medians with interquartile ranges in brackets.

- **Leaf color** Reflects the *cNFC* most strongly linked to the indicator. In some cases, the assigned color may differ from the one with the highest correlation.
- **Labels:** Show abbreviated names of the WDI indicators for clarity.

This structure allows for mapping *cNFC* components back to interpretable thematic clusters in the WDI, helping contextualize the dimensionality-reduced representations used in the main model.

S6: Geographical Patterns and Shared Development Histories

The spatial distribution of the *cNFCs* shows that structural development trajectories are shaped by geography and history. As seen in supplement figure S4, countries form clear regional clusters within the latent space. Former Soviet and Eastern European states occupy a compact area characterized by negative values along *cNFC 2*, reflecting industrially dependent or state-controlled economic structures. Latin American countries cluster along low values of *cNFC 4*, which represent mixed-service economies and demographic pressures linked to public health constraints. Sub-Saharan African countries are located toward lower values of *cNFC 1*, corresponding to lower-income, agrarian, and demographically young contexts. High-income countries in Europe and North America occupy the upper range of *cNFC 1*, consistent with advanced service economies and aging populations. These regional patterns show that the socioeconomic and institutional conditions associated might be shaped by historical and geographical contexts. The clustering of countries with similar development paths, such as those following post-Soviet transitions or Latin American industrialization, suggests that risk and resilience are associated with long-term structural trajectories. By capturing these relationships, the *cNFCs* help explain how historically grounded development

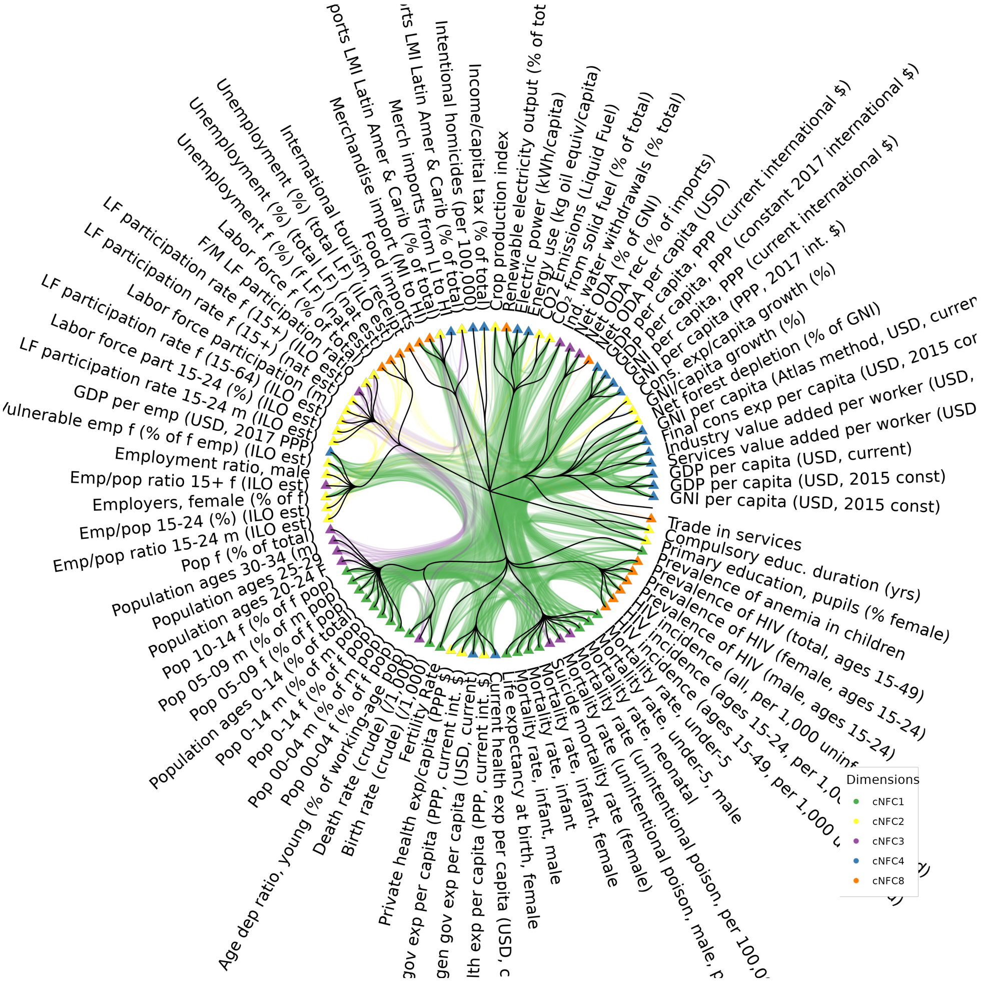

Fig. S3 World bank indicators associated with the *cNFCs*. Indicators with the highest associations with the *cNFC* shown on the outer circle. The number of included parameters is proportional the prediction of excess mortality. Every line is associated with a WDI. In the black centre dendrogram, the thematically order of the world bank is shown.

structures continue to influence national coping capacity and the unequal health outcomes observed during the pandemic. This illustration is an updated version of the already published finding by Kraemer et al 2020 [2].

S7: Predictive performance of the integrated model

The integrated model incorporates the three components defined in the Methods section. To obtain robust estimates of predictive performance, we ran the regression trees 1,000 times using identical hyperparameter constraints across all runs. The model

was restricted by allowing only 50 or 100 boosting rounds, limiting the maximum tree depth to 2 or 3, and fixing the learning rate to 0.05 (see the published script on GitHub for further details). These settings were chosen to reduce the risk of overfitting and to ensure a consistent comparison across runs.

Despite this conservative tuning, the distribution of explained variance across runs remains relatively wide (Supplementary figure S5).

However, the error distribution is symmetric and centered around a stable mean, suggesting that the observed variation is unlikely to be driven by systematic overfitting or underfitting. Instead, it likely reflects differences in the composition of the randomly drawn test sets. To explore this possibility, we examined which countries appeared in test sets associated with higher or lower explained variance. We found no significant association between the Income level and the mean error of a run (Script: 04_plot_outline S7).

We further validated our modeling approach by repeating the analysis using both linear regression and random forest models [3]. As expected, the linear models performed substantially worse, achieving an average explained variance of only $R^2 = 24.7\%$ across 50 runs. In contrast, the random forest approach performed considerably better, reaching $R^2 = 40.2\%$.

These results suggest that the relationships in the data are strongly non-linear, and that even less flexible machine-learning methods such as random forests can capture a meaningful share of the explained variance, although still below the performance of XGBoost. However, because random forests provide lower predictive power and do not allow the same granular hyper parameter tuning, we do not rely on them in the main results. Additional details of these robustness checks are provided in Script: 04_plot_outline (Q3).

References

[1] Tenenbaum, J.B., Silva, V.d., Langford, J.C.: A global geometric framework for nonlinear dimensionality reduction. science **290**(5500), 2319–2323 (2000)

[2] Kraemer, G., Reichstein, M., Camps-Valls, G., Smits, J., Mahecha, M.D.: The Low Dimensionality of Development. Social Indicators Research (2020) https://doi.org/10.1007/s11205-020-02349-0

[3] Ronco, M., Tárraga, J.M., Muñoz, J., Piles, M., Marco, E.S., Wang, Q., Espinosa, M.T.M., Ponserre, S., Camps-Valls, G.: Exploring interactions between socioeconomic context and natural hazards on human population displacement. Nature Communications **14**(1), 8004 (2023) https://doi.org/10.1038/s41467-023-43809-8 . Accessed 2024-06-20

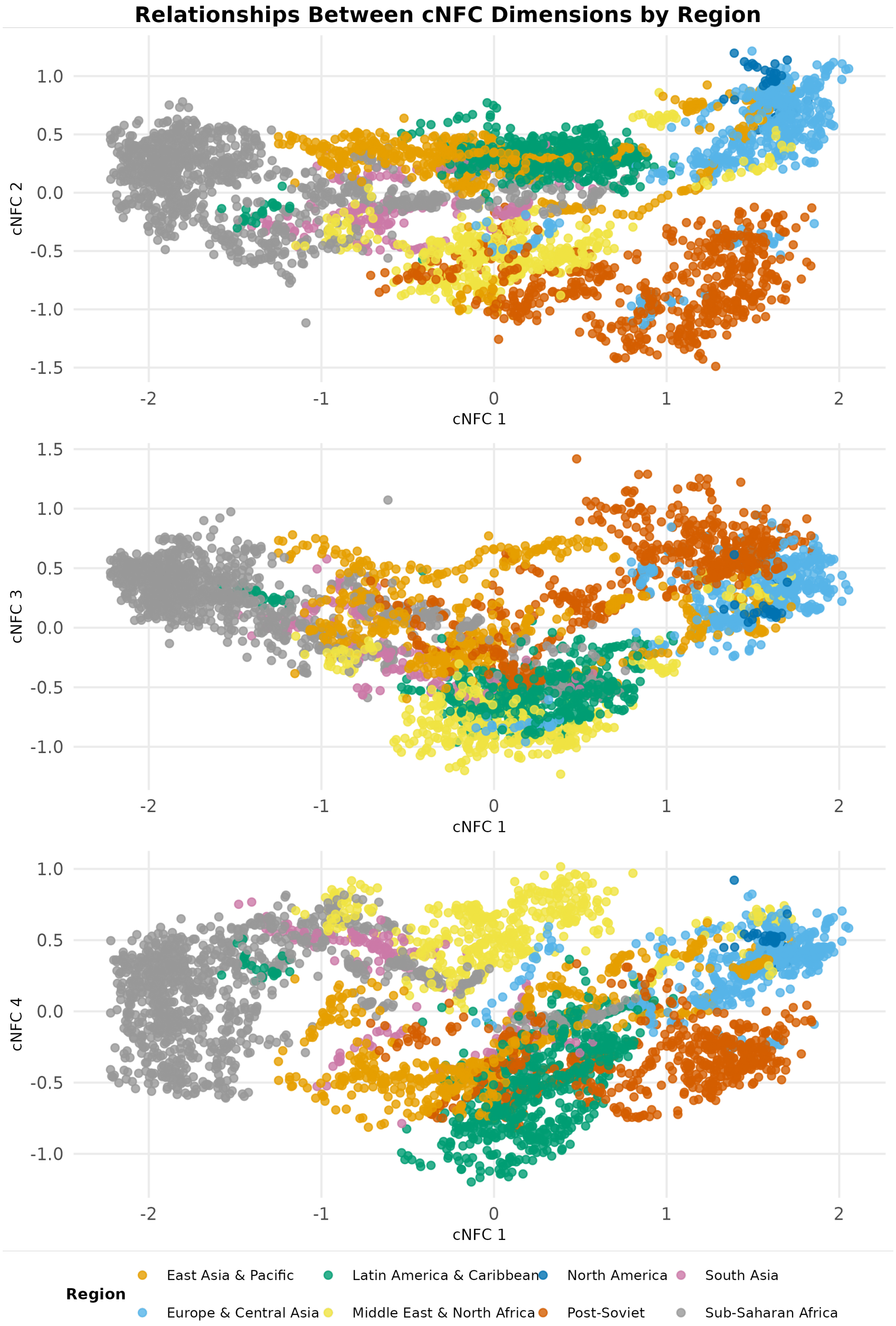

Fig. S4 Latent socioeconomic space colored by world regions. Each point represents one country–year between 1990 and 2019. The three panels show the relationships between *cNFC 1* and *cNFC 2*, *cNFC 3*, and *cNFC 4*. Regional clustering illustrates shared historical and developmental trajectories that shape the structural composition of the *cNFCs*

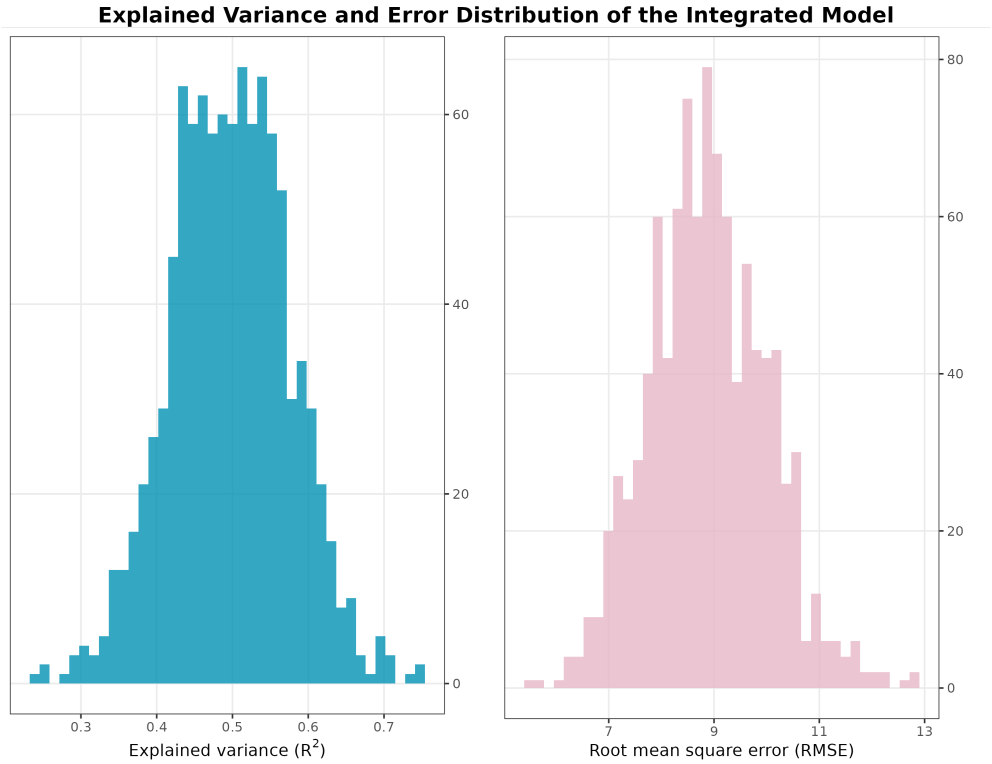

Fig. S5 Histogram of summary statistics from the integrated model, showing the distribution of explained variance and prediction error across 1,000 runs.
